# First-in-human evaluation of 6-bromo-7-[^11^C]methylpurine, a PET tracer for assessing the function of multidrug resistance-associated proteins in different tissues

**DOI:** 10.1007/s00259-024-06851-2

**Published:** 2024-07-26

**Authors:** Severin Mairinger, Matthias Jackwerth, Zacharias Chalampalakis, Ivo Rausch, Maria Weber, Michael Wölfl-Duchek, Lena Pracher, Lukas Nics, Jens Pahnke, Werner Langsteger, Marcus Hacker, Markus Zeitlinger, Oliver Langer

**Affiliations:** 1 Department of Clinical Pharmacology, Medical University of Vienna, Vienna, Austria; 2 Department of Biomedical Imaging and Image-guided Therapy, Medical University of Vienna, Vienna, Austria; 3 QIMP Team, Center for Medical Physics and Biomedical Engineering, Medical University of Vienna, Vienna, Austria; 4 Translational Neurodegeneration Research and Neuropathology Lab, Department of Clinical Medicine (KlinMed), Medical Faculty, University of Oslo, Oslo, Norway; 5 Section of Neuropathology Research, Department of Pathology, Clinics for Laboratory Medicine (KLM), Oslo University Hospital, Oslo, Norway; 6 Institute of Nutritional Medicine (INUM) and Lübeck Institute of Dermatology (LIED), University of Lübeck and University Medical Center Schleswig-Holstein, Lübeck, Germany; 7 Department of Pharmacology, Faculty of Medicine and Life Sciences, University of Latvia, Rīga, Latvia; 8 School of Neurobiology, Biochemistry and Biophysics, The Georg S. Wise Faculty of Life Sciences, Tel Aviv University, Tel Aviv, Israel

**Keywords:** Long axial field-of-view PET/CT, 6-Bromo-7-[^11^C]methylpurine, Multidrug resistance-associated proteins, MRP1, Test-retest variability, Dosimetry

## Abstract

**Purpose:**

Multidrug resistance-associated protein 1 (MRP1) is a transport protein with a widespread tissue distribution, which has been implicated in the pathophysiology of Alzheimer’s and chronic respiratory disease. PET with 6-bromo-7-[^11^C]methylpurine ([^11^C]BMP) has been used to measure MRP1 function in rodents. In this study, [^11^C]BMP was for the first time characterised in humans to assess the function of MRP1 and other MRP subtypes in different tissues.

**Methods:**

Thirteen healthy volunteers (7 men, 6 women) underwent dynamic whole-body PET scans on a long axial field-of-view (LAFOV) PET/CT system after intravenous injection of [^11^C]BMP. Three subjects of each sex were scanned a second time to assess reproducibility. Volumes of interest were outlined for MRP-expressing tissues (cerebral cortex, cerebellum, choroid plexus, retina, lungs, myocardium, kidneys, and liver). From the time-activity curves, the elimination rate constant (*k*_E_, h^− 1^) was derived as a parameter for tissue MRP function and its test-retest variability (TRTV, %) was calculated. Radiation dosimetry was calculated using the Medical Internal Radiation Dose (MIRD) methodology.

**Results:**

Mean *k*_E_ and corresponding TRTV values were: cerebral cortex: 0.055 ± 0.010 h^− 1^ (− 4 ± 24%), cerebellum: 0.033 ± 0.009 h^− 1^ (1 ± 39%), choroid plexus: 0.292 ± 0.059 h^− 1^ (0.1 ± 16%), retina: 0.234 ± 0.045 h^− 1^ (30 ± 38%), lungs: 0.875 ± 0.095 h^− 1^ (− 3 ± 11%), myocardium: 0.641 ± 0.105 h^− 1^ (11 ± 25%), kidneys: 1.378 ± 0.266 h^− 1^ (14 ± 16%), and liver: 0.685 ± 0.072 h^− 1^ (7 ± 9%). Significant sex differences were found for *k*_E_ in the cerebellum, lungs and kidneys. Effective dose was 4.67 ± 0.18 µSv/MBq for men and 4.55 ± 0.18 µSv/MBq for women.

**Conclusion:**

LAFOV PET/CT with [^11^C]BMP potentially allows for simultaneous assessment of MRP function in multiple human tissues. Mean TRTV of *k*_E_ in different tissues was in an acceptable range, except for the retina. The radiation dosimetry of [^11^C]BMP was in the typical range of ^11^C-tracers. LAFOV PET/CT holds great potential to assess at a whole-body, multi-tissue level molecular targets relevant for drug disposition in humans.

**Trial registration:**

EudraCT 2021-006348-29. Registered 15 December 2021.

**Supplementary Information:**

The online version contains supplementary material available at 10.1007/s00259-024-06851-2.

## Introduction

Multidrug resistance-associated protein 1 (MRP1, encoded in humans by the *ABCC1* gene and in rodents by the *Abcc1* gene) is an adenosine triphosphate-binding cassette (ABC) transporter with a widespread tissue distribution [1, 2]. MRP1 transports a broad range of endogenous and exogenous compounds, including drugs and drug metabolites. MRP1 is overexpressed in some tumour types and contributes to multidrug resistance by exporting a range of anticancer drugs from tumour cells (e.g., vincristine, doxorubicin, epirubicin, etoposide, daunorubicin, and mitoxantrone) [2]. MRP1 has been further implicated in the pathophysiology of Alzheimer’s disease (AD) [3] and chronic respiratory disease, such as chronic obstructive pulmonary disease [4] and cystic fibrosis [5]. There is evidence that MRP1 contributes to the brain clearance of neurotoxic amyloid-beta (Aβ) peptides across the blood-brain barrier (BBB) and the blood-cerebrospinal fluid barrier [3]. Pharmacological stimulation of MRP1 function has been proposed as a therapeutic strategy to promote brain Aβ clearance in AD patients [3]. To translate such treatment approaches to humans and to assess the role of MRP1 in different diseases, there is a need for methodology to measure MRP1 function in vivo. While several positron emission tomography (PET) radiotracers are available to measure the function of P-glycoprotein (P-gp/ABCB1), another important ABC transporter [6], MRP1 function has so far not been assessed with PET in humans.

6-Bromo-7-[^11^C]methylpurine ([^11^C]BMP) has been used to measure MRP1 function in the brain and lungs of mice and rats with PET [7–14]. Following intravenous (i.v.) injection, [^11^C]BMP is efficiently delivered to different tissues by passive diffusion, where it is rapidly converted by intracellular glutathione-*S*-transferase enzymes (GSTs) into its glutathione (GSH) conjugate *S*-(6-(7-[^11^C]methylpurinyl))glutathione ([^11^C]MPG) [7, 8]. Due to its high polarity, [^11^C]MPG cannot diffuse back into blood and is eliminated from tissues by MRP1 and possibly other MRP subtypes [9, 15]. At the same time, [^11^C]BMP is also rapidly converted in blood into [^11^C]MPG. The rate constant for radioactivity elimination from tissues (*k*_E_), which can be directly derived from the tissue TACs without the need to consider blood radioactivity, has been validated as a parameter for tissue MRP1 function in rodent studies [7–9]. In vitro data indicate that MPG is also efficiently transported by human MRP1 [12, 16].

Given the widespread tissue distribution of MRP1 and other MRP subtypes, whole-body dynamic imaging would be desirable to assess MRP function with [^11^C]BMP and PET. While this is feasible with small-animal PET imaging in mice [9, 10, 13], the limited axial field of view (FOV) of previously available clinical PET scanners has hampered a simultaneous assessment of transporter function in multiple tissues in humans.

In the present study, [^11^C]BMP was for the first time characterised in humans, employing a long axial field-of-view (LAFOV) PET/CT system [17], for the quantification of MRP function at a whole-body level. We report test-retest variability (TRTV) data in healthy volunteers, sex differences in tissue MRP function and the human dosimetry of [^11^C]BMP.

## Materials and methods

### General

This study was conducted in accordance with the ICH-GCP guidelines and the Declaration of Helsinki. The trial was registered in the EudraCT database (2021-006348-29) as a phase 1 first-in-human study and was approved by the Ethics Committee of the Medical University of Vienna and the Austrian Agency for Health and Food Safety. To obtain regulatory approval for the study, an investigational medicinal product dossier (IMPD) containing non-clinical toxicity data and human dosimetry estimates was prepared for [^11^C]BMP as described before [18]. All subjects gave oral and written informed consent before enrolment in the study. Thirteen healthy subjects (7 men: age: 28 ± 2 years, weight: 80 ± 13 kg and 6 women: age: 26 ± 1 years, weight: 60 ± 10 kg) were included into our study. Subjects were free of any medication for at least 14 days and judged as healthy based on clinical examination and routine blood and urine laboratory assessments.

### Radiotracer synthesis

[^11^C]BMP was automatically synthesised in a TRACERlab™ FX2 C synthesis module (GE Healthcare, Uppsala, Sweden) by regioselective *N*^*7*^-methylation of 6-bromo-7 H-purine with [^11^C]methyl triflate as described before [18]. [^11^C]BMP was formulated in 0.9% aqueous sodium chloride solution and ethanol (9/1, *v*/*v*). For i.v. injection into humans, an aliquot of the formulated product solution was further diluted with 0.9% aqueous sodium chloride solution to a final injection volume of 10 ml.

### PET/CT imaging

All subjects (7 men, 6 women) underwent single dynamic whole-body PET scans after i.v. injection of [^11^C]BMP on a Biograph Vision Quadra PET/CT system (Siemens Healthineers, Knoxville, TN, USA) (axial FOV: 106 cm) [17]. To assess TRTV, three subjects of each sex were scanned a second time with a mean time interval between the test and the retest scan of 20 ± 10 days (range: 7–28 days). Subjects were scanned in supine position with arms down. First, a low-dose computed tomography (CT) scan (CareDose4D, CarekV setting: Semi, ref. tube voltage: 100 kVp with Sn filter, ref. tube current: 30 mAs) was acquired for attenuation correction. Then, [^11^C]BMP (368 ± 18 MBq, containing < 50 µg of unlabelled BMP) was administered as an i.v. bolus over 20 s. At the start of the injection, a 90-min list mode PET acquisition was started.

### Blood and urine sampling

Venous blood samples were manually collected *via* a peripheral venous catheter in an antecubital vein at 5, 10, 20, 30, 40, 60, and 90 min after radiotracer injection. The first 2 ml of each blood draw were discarded. Radioactivity in blood and plasma aliquots was measured in a gamma counter (Perkin Elmer 1480 Wizard 3 gamma counter, Meriden, CT, USA), which had been cross-calibrated with the PET/CT scanner. The plasma samples collected at 5, 20, and 40 min after radiotracer injection were analysed with radio-high performance liquid chromatography (radio-HPLC) to assess conversion of [^11^C]BMP into [^11^C]MPG as described in the following section. After the imaging session, subjects were asked to empty their urinary bladder. Radioactivity in urine aliquots was measured in the gamma counter. An undiluted 2-ml urine sample was injected into the same radio-HPLC system as used for the plasma analysis to assess the percentage of [^11^C]MPG in urine. Decay-corrected urinary radioactivity concentrations were multiplied by the collected urine volume to obtain the percentage of the administered activity excreted into the urine.

### Plasma metabolite analysis

Plasma samples (830 µl) were mixed with acetonitrile (600 µl) and vortexed to precipitate plasma proteins. After addition of water (600 µl) and phosphate-buffered saline (10-fold concentrate, pH 7.4, 100 µl), samples were centrifuged (4 min, 15,000 × g, 4 °C). For the 5 min sample, the protein pellet and supernatant were separately counted in the gamma counter to determine the recovery of radioactivity. The supernatant (2 ml) was then spiked with unlabelled BMP (0.4 mg/ml in water, 50 µl) and unlabelled MPG (1.0 mg/ml in water, 50 µl) and injected into the radio-HPLC system. An Atlantis T3 OBD HPLC column (250 × 10 mm, 10 μm, Waters, Austria) equipped with a pre-column (Atlantis T3 Prep Guard Cartridge, 10 × 10 mm, 10 μm, Waters, Austria) was eluted with a mixture of 25 mM aqueous ammonium acetate (solvent A) and acetonitrile (solvent B). First, a linear gradient from 5 to 15% of solvent B over 7 min was applied to the column, followed by a step gradient to 35% of solvent B with a total run time of 14 min and a flow rate of 5 ml/min. On this HPLC system, [^11^C]BMP and [^11^C]MPG eluted with retention times of approximately 11.2 min and 3.3 min, respectively. HPLC eluates were collected in 1-min fractions, which were counted in the gamma counter. The measured fractions were corrected for radioactive decay to determine the percentages of [^11^C]BMP and [^11^C]MPG in plasma at different time points.

### MR imaging

On a separate day after completion of the PET/CT examination, a T1-weighted brain magnetic resonance imaging (MRI) scan was acquired in each subject on a Siemens Magnetom Skyra 3T MR system with the following parameters: echo time/repetition time = 2.32/1730 ms, inversion time = 942 ms, flip angle = 10°, 240 × 240 mm field of view, 176 slices, voxel size: 0.47 × 0.47 × 0.9 mm.

### Image analysis

The PET list mode data were re-binned into 1 × 15 s, 3 × 5 s, 3 × 10 s, 2 × 30 s, 3 × 60 s, 2 × 150 s, 2 × 300 s, and 7 × 300 s frames and each PET frame was reconstructed into a 440 × 440 × 531 matrix (voxel size: 1.65 × 1.65 × 2 mm^3^) with an ordinary Poisson ordered subset expectation maximisation algorithm (OP-OSEM, 4 iterations, 5 subsets) with PSF modelling and TOF information. A 2 mm FWHM Gaussian post-reconstruction filter was applied to all images. Scatter correction, CT-attenuation correction and dead-time and randoms correction were applied to the PET data. Volumes of interest (VOIs) were manually outlined on the co-registered PET/CT data for the right lung, myocardium, right kidney cortex, liver, urinary bladder, and gall bladder (including the extrahepatic bile duct) in the PFUS tool in PMOD (version 4.404, PMOD Technologies Ltd., Zürich, Switzerland) (Supplementary Fig. 1). In case of subjects’ motion, the position of the different VOIs was manually adjusted in the individual PET time frames. As the gall bladder was not always clearly visible on the PET images it could not be outlined in all subjects. Three spherical VOIs (diameter: 10 mm) were placed in the right lung (one in each lobe) and averaged to generate a global lung VOI. Three spherical VOIs (diameter: 10 mm) were placed in the liver (two in the right and one in the left liver lobe) and averaged to generate a global liver VOI. One spherical VOI (diameter: 8 mm) was placed in the right kidney cortex. One spherical VOI (diameter: 6 mm) was placed in the myocardium. For the urinary bladder and the gall bladder, the VOIs included all the visible radioactivity.

The brain kinetics of [^11^C]BMP were analysed using a brain region atlas (N30R83) implemented in the PNEURO tool in PMOD. Brain PET data were automatically motion-corrected in the PNEURO tool. The anatomical MRI was segmented into grey and white matter and spatially normalised to a Montreal Neurological Institute (MNI) T_1_-MRI template before transferring the atlas regions to the PET data. For a preliminary analysis of the brain distribution of radioactivity, we selected among the 83 available regions from the brain atlas a global cortical grey matter VOI, composed of all cortical sub-regions, and a cerebellar grey matter VOI because of the clearly visible difference in radioactivity concentrations between these two regions (Supplementary Fig. 2a). The choroid plexus and the retina were manually outlined on the MRI data using the PNEURO tool (Supplementary Fig. 2b, c). For the choroid plexus, four spherical VOIs (diameter: 2 mm) were placed in the first and second ventricle (two in each hemisphere) and averaged to generate a global choroid plexus VOI (Supplementary Fig. 2b). From the VOIs, time-activity curves (TACs) in units of standardised uptake value (SUV) were extracted. For selected MRP-expressing tissues (i.e., cerebral cortex, cerebellum, choroid plexus, retina, lungs, myocardium, kidneys and liver), the rate constant (*k*_E_) for radioactivity elimination was determined as a parameter for tissue MRP function [7–9]. *k*_E_ is equivalent to the fraction of radioactivity that is eliminated from tissue per time and has a unit of h^− 1^. *k*_E_ equals the slope of the linear part of the natural logarithm-transformed tissue TACs from 15 to 90 min after radiotracer injection and was obtained by linear regression analysis using Microsoft^®^ Excel^®^ 2019 MSO. It should be noted that *k*_E_ is not identical with the efflux rate constant from tissue into plasma *k*_2_ determined with compartmental modelling, which requires the measurement of an arterial plasma input function.

### Dosimetry

For the dosimetry calculations, VOIs were generated using an Artificial Intelligence segmentation tool, multiple-organ objective segmentation (MOOSE), and validated using visual inspection [19]. The considered organs and tissues were brain, thyroid gland, right lung, myocardium, liver, gall bladder, kidneys, red bone marrow (L3 to L5), muscle (gluteus maximus), and urinary bladder. PET frame images were analysed using an in-house pipeline (built using the Python programming language) to extract TACs for each region. Absorbed doses were calculated using the Medical Internal Radiation Dose (MIRD) methodology [20] with the MIRDcalc software for organ level dosimetry [21, 22]. The standard adult male and female International Commission on Radiological Protection (ICRP) reference phantoms were used for organ masses and source to target organ dose values (S values). Time-integrated activity in the source regions was calculated by integration over the measured time points, while assuming radioactive decay after the 90-min time point. Those were then normalised to each subject’s injected activity to calculate time-integrated-activity coefficients (residence times) and input to MIRDcalc. The sampled organs’ contribution to the total residence time was 37 ± 7% with the remaining being attributed to the rest of the body.

### Statistical analysis

Descriptive data are presented as mean ± standard deviation (SD) unless otherwise specified. Reproducibility of *k*_E_ was assessed using the TRTV (%), which was calculated for each VOI in each subject as ((*k*_E, test_ − *k*_E, retest_) / (*k*_E, test_ + *k*_E, retest_) / 2) × 100%. Differences between test and retest scans were analysed using a two-sided, paired t-test. Sex differences were analysed using a two-sided, unpaired t-test. The level of statistical significance was set to a *p* value of ≤ 0.05. Statistical analysis was performed using Prism 10.2.3 (Graphpad Software, Dotmatics, Boston, MA, USA).

## Results

**Fig. 1:**
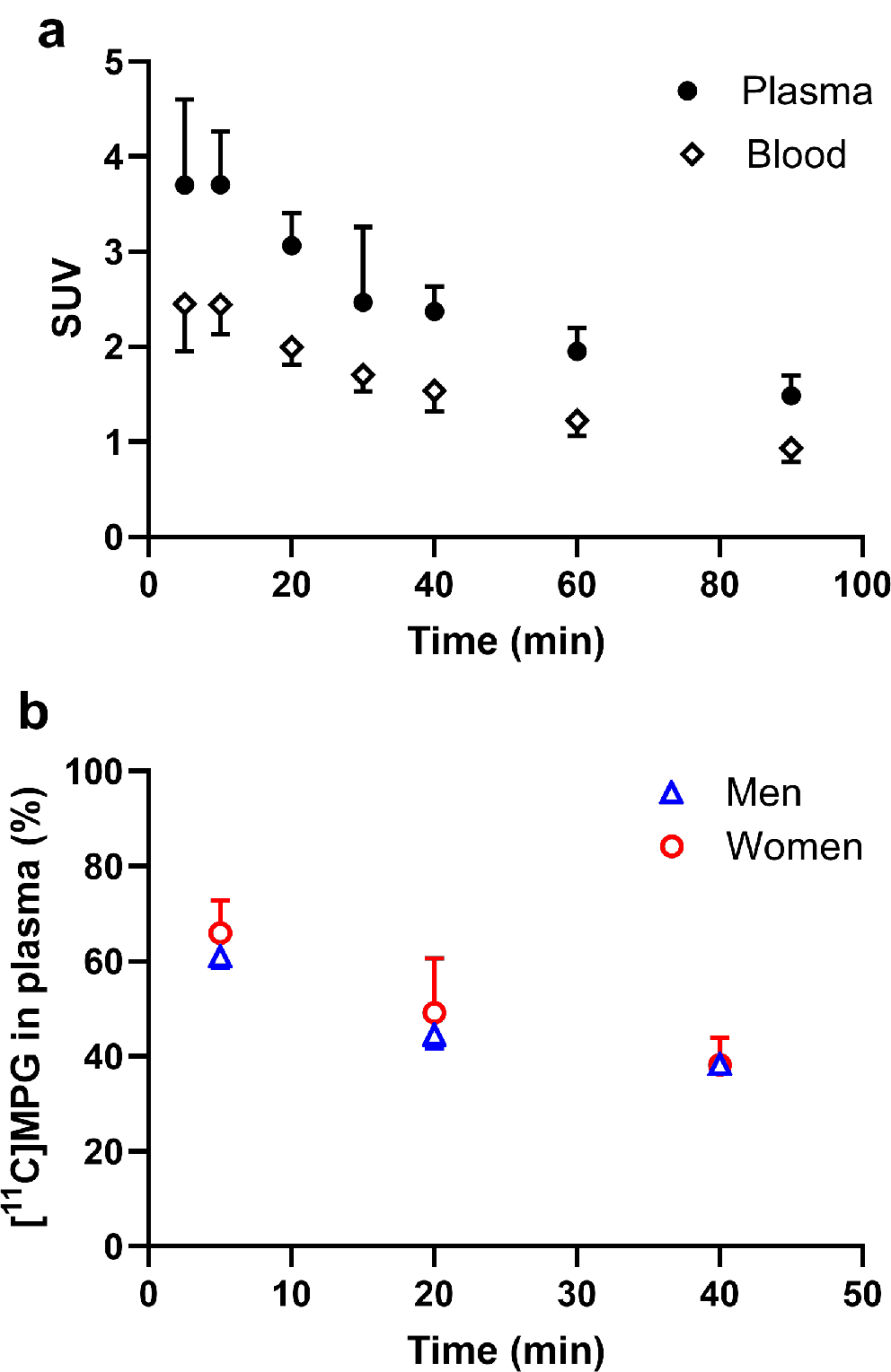
Mean (SUV ± SD, *n* = 13) time-activity curves of total radioactivity in venous blood and plasma (**a**) and percentage of the radiolabelled glutathione conjugate ([^11^C]MPG) in venous plasma of male (*n* = 7) and female (*n* = 6) subjects (**b**)

**Fig. 2:**
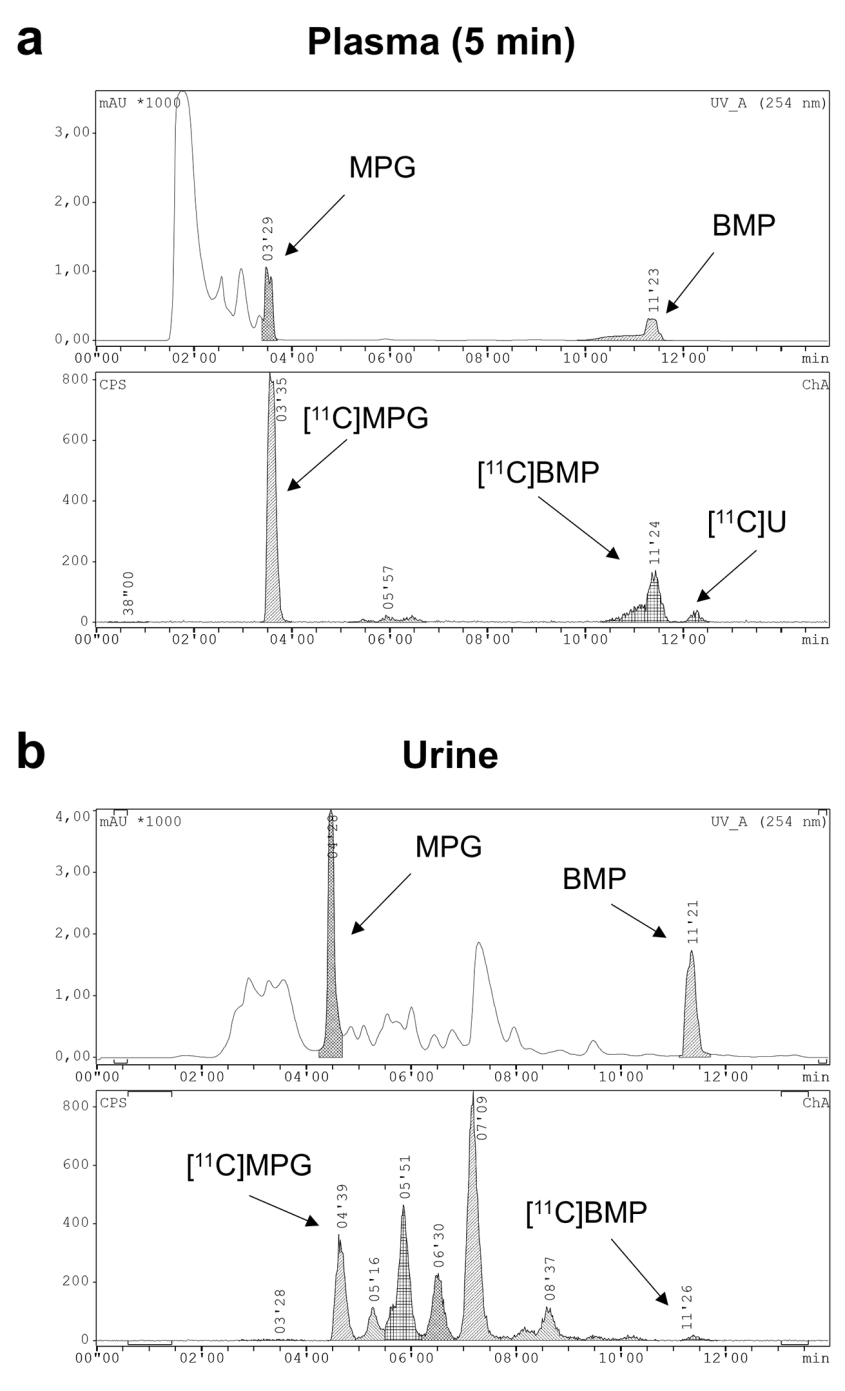
Representative HPLC chromatograms for analysis of plasma collected at 5 min after [^11^C]BMP injection (**a**) and urine collected at the end of the PET scan (**b**). The upper channel represents UV absorption (254 nm) and the lower channel radioactivity detection. Samples were spiked with unlabelled BMP and MPG. Retention times were different between plasma and urine due to matrix differences. [^11^C]U designates an unidentified radiolabelled species detected in plasma

We included 13 subjects (7 men, 6 women) into our study, who underwent dynamic whole-body PET scans on a LAFOV PET/CT system after i.v. injection of [^11^C]BMP. Radiotracer administration was well tolerated without occurrence of adverse effects. During the PET scan venous blood samples were collected. Figure 1a shows mean TACs of total radioactivity in venous blood and plasma. Plasma-to-blood ratios of activity gradually increased over the duration of the PET scan (10 min: 1.51 ± 0.09; 90 min: 1.59 ± 0.08, *p* ≤ 0.001). There were significant sex differences in plasma-to-blood ratios, both at the 10 min time point (men: 1.59 ± 0.06, women: 1.44 ± 0.05, *p* ≤ 0.001) and at the 90 min time point (men: 1.65 ± 0.06, women: 1.52 ± 0.03, *p* ≤ 0.001). Plasma samples obtained at 5, 20, and 40 min after radiotracer injection were extracted with acetonitrile and analysed with radio-HPLC to assess conversion of [^11^C]BMP into its GSH conjugate [^11^C]MPG (Fig. 2a). The mean recovery for extraction of radioactivity into the acetonitrile fraction was 92.8 ± 1.4% (*n* = 18) for the 5 min sample. [^11^C]MPG and unconverted [^11^C]BMP were the two major radiolabelled species detected in plasma (Fig. 2a). In addition, an unidentified, lipophilic radiolabelled species, which eluted after [^11^C]BMP, was observed. The percentages of the three radiolabelled species in plasma at different time points are summarised in Supplementary Table 1. The percentage of unconverted [^11^C]BMP in plasma remained relatively constant (approximately 30% of total radioactivity), while the percentage of the GSH conjugate [^11^C]MPG declined from 63 ± 5% at 5 min to 38 ± 4% at 40 min and the percentage of the unidentified, lipophilic radiolabelled species increased over time (Supplementary Table 1). There were no sex differences in the percentage of [^11^C]MPG in plasma at all studied time points (Fig. 1b).

**Fig. 3:**
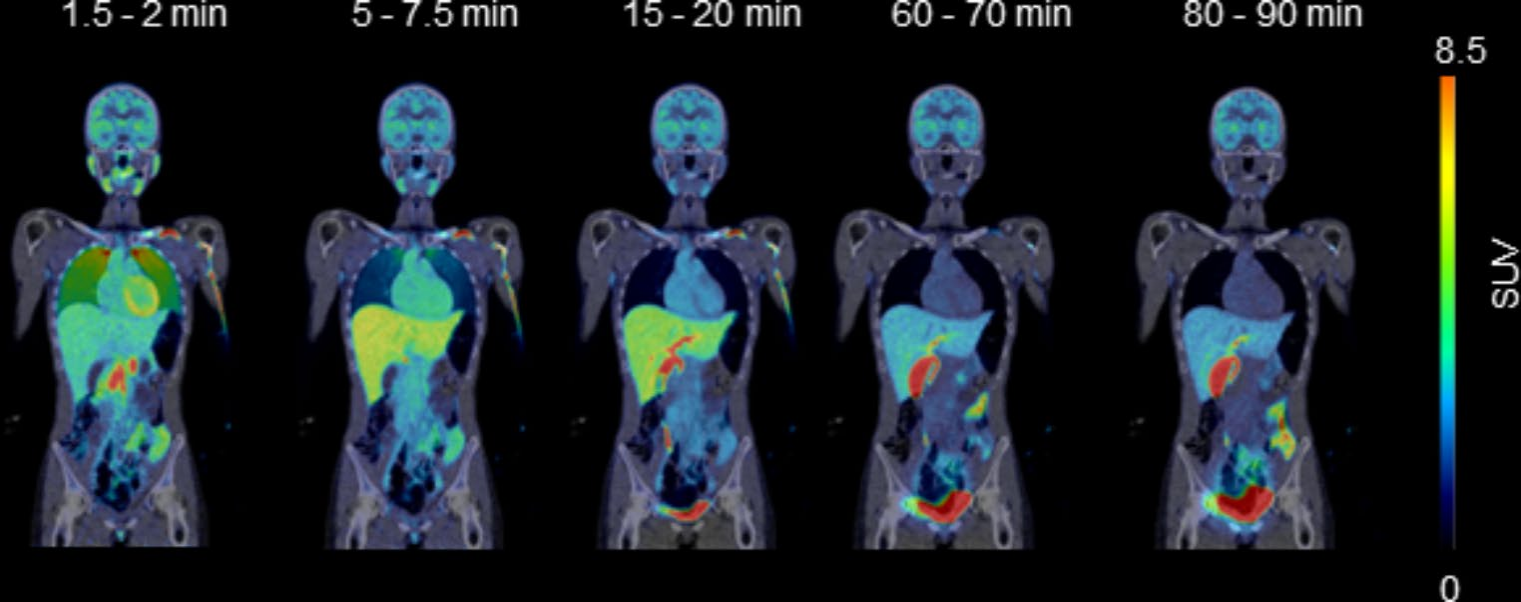
Coronal whole-body PET/CT images obtained on the LAFOV scanner (axial FOV: 106 cm) at different time points after injection of 389 MBq [^11^C]BMP in one representative female subject (25 years, 66 kg). Activity scale is given in SUV units and set from 0 to 8.5

**Fig. 4:**
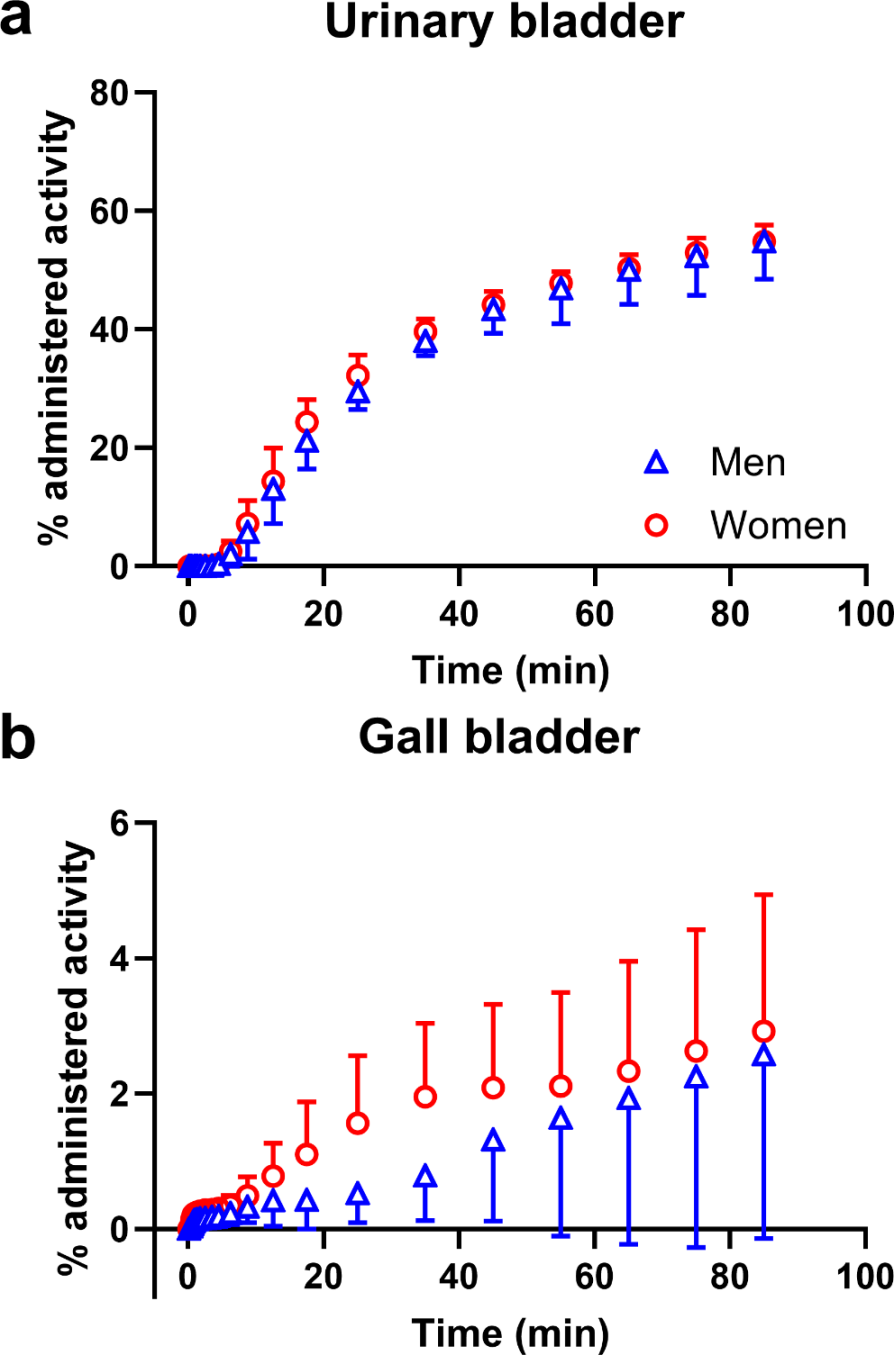
Mean (percentage of the administered activity ± SD) time-activity curves for the excretion of [^11^C]BMP-derived radioactivity into the urinary bladder (**a**) and the gall bladder (**b**) of male (*n* = 7, gall bladder: *n* = 5) and female (*n* = 6, gall bladder: *n* = 4) subjects

Figure 3 shows representative whole-body PET/CT images obtained in one female subject at different time points after injection of [^11^C]BMP. Over the time course of the PET scan, activity was predominantly excreted into the urinary bladder. Figure 4a shows TACs for activity excretion into the urinary bladder. The percentage of the administered activity excreted into the urinary bladder at the end of the PET scan was 55 ± 5% based on the urinary bladder VOI delineated on the PET images and 51 ± 11% based on the gamma counter measurements of urine collected at the end of the PET scan. There were no significant sex differences in the percentage of the administered activity excreted into the urinary bladder (Fig. 4a). Radio-HPLC analysis of urine samples revealed a mixture of several, partly unidentified radiolabelled species with only very low amounts of unconverted [^11^C]BMP (< 1%) (Fig. 2b). The GSH conjugate [^11^C]MPG represented 26 ± 7% of total radioactivity in the urine. The amount of activity excreted into the gall bladder was rather low and variable (2.7 ± 2.3% of the administered activity) (Fig. 4b).

**Fig. 5:**
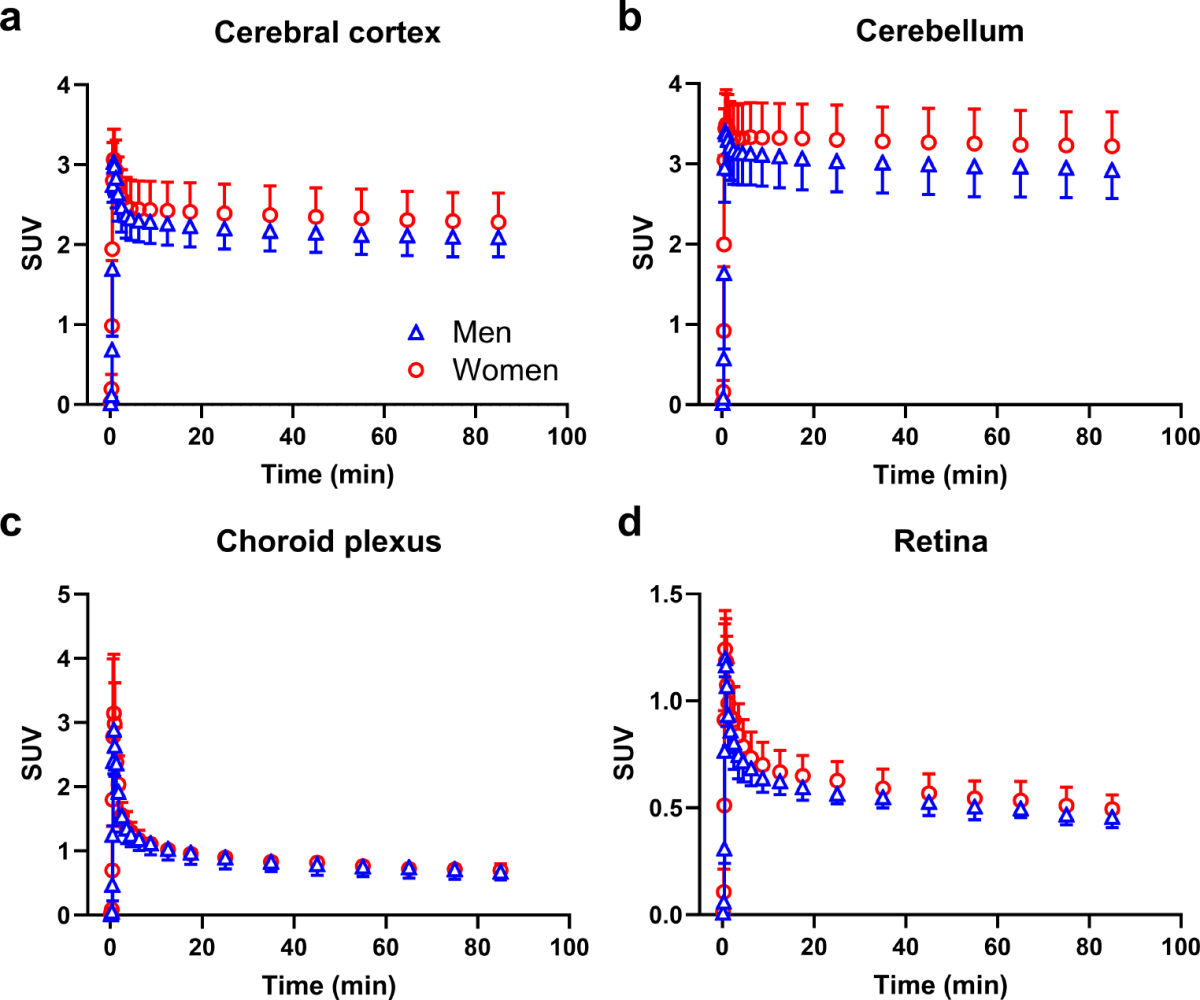
Mean (SUV ± SD) time-activity curves in central MRP-expressing tissues, i.e., cerebral cortex (**a**), cerebellum (**b**), choroid plexus (**c**), and retina (**d**), of male (*n* = 7) and female (*n* = 6) subjects

**Fig. 6:**
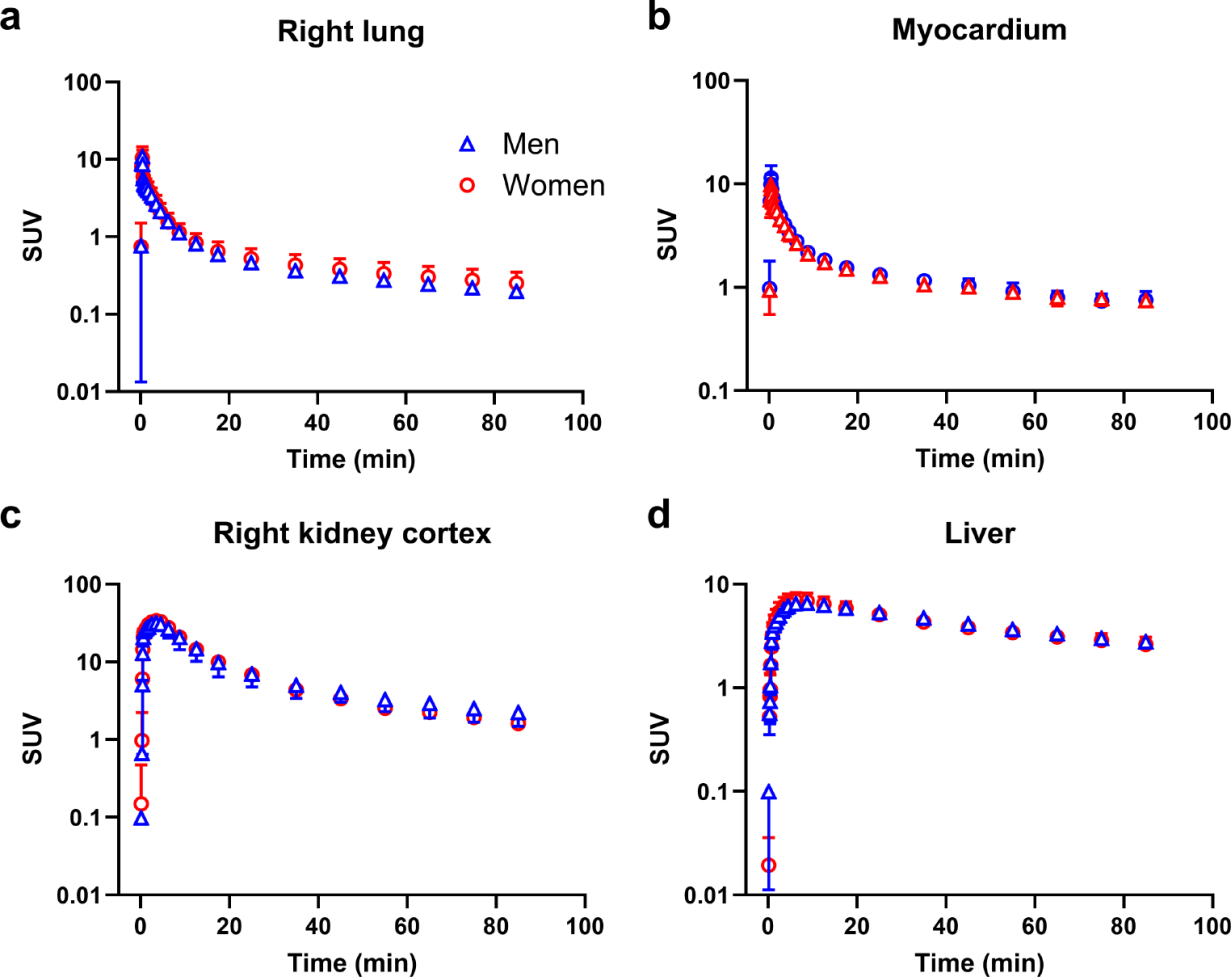
Mean (SUV ± SD) time-activity curves in peripheral MRP-expressing tissues, i.e., right lung (**a**), myocardium (**b**), right kidney cortex (**c**), and liver (**d**), of male (*n* = 7) and female (*n* = 6) subjects

We outlined VOIs for different MRP-expressing tissues. The corresponding TACs in male and female subjects are shown in Fig. 5 for central tissues and in Fig. 6 for peripheral tissues. We observed regional differences in the brain distribution of [^11^C]BMP-derived radioactivity with a significantly higher exposure in the cerebellum than in the cortex (area under the TAC, cerebellum: 265 ± 34 SUV × min, cortex: 192 ± 26 SUV × min, *p* ≤ 0.0001) (Fig. 5a, b, Supplementary Fig. 2a). As an outcome parameter for tissue MRP function we derived *k*_E_ from the tissue TACs. Mean *k*_E_ values for all investigated tissues are given in Table 1. The slowest activity elimination was observed for the cerebellum (*k*_E_ = 0.033 ± 0.009 h^− 1^) and the fasted elimination for the kidneys (*k*_E_ = 1.378 ± 0.266 h^− 1^). There were significant sex differences in *k*_E_ for the cerebellum (*p* ≤ 0.01), the lungs (*p* ≤ 0.05), and the kidneys (*p* ≤ 0.05) (Table 1). In the brain, *k*_E_ was significantly lower for the cerebellum than for the cortex (*p* ≤ 0.0001).

**Table 1:** *k*_E_ values in different tissues as a measure of MRP function

Tissue	k_E_ (h^− 1^) ^a^	TRTV (%) ^b^	k_E_ men (h^− 1^) ^c^	k_E_ women (h^− 1^) ^d^
Cerebral cortex	0.055 ± 0.010	−4 ± 24	0.057 ± 0.009	0.051 ± 0.011
Cerebellum	0.033 ± 0.009	1 ± 39	0.038 ± 0.006	0.027 ± 0.008 ^*^
Choroid plexus	0.292 ± 0.059	0.1 ± 16	0.298 ± 0.040	0.284 ± 0.079
Retina	0.234 ± 0.045	30 ± 38	0.230 ± 0.059	0.240 ± 0.025
Right lung	0.875 ± 0.095	−3 ± 11	0.928 ± 0.075	0.813 ± 0.080 ^*^
Myocardium	0.641 ± 0.105	11 ± 25	0.606 ± 0.108	0.682 ± 0.093
Right kidney cortex	1.378 ± 0.266	14 ± 16	1.238 ± 0.261	1.542 ± 0.166 ^*^
Liver	0.685 ± 0.072	7 ± 9	0.675 ± 0.069	0.698 ± 0.081

^a^ Mean *k*_E_ value of all subjects (7 men and 6 women, not including retest scans)

^b^ Test-retest variability calculated from data in 3 men and 3 women

^c^*n* = 7 men

^d^*n* = 6 women

^*^ Significantly different from men (cerebellum: *p* ≤ 0.01; right lung and right kidney cortex: *p* ≤ 0.05, two-sided, unpaired t-test)

Three subjects of each sex were scanned a second time to assess the TRTV of *k*_E_. In none of the investigated tissues were *k*_E_ values significantly different between the test and the retest scans (Supplementary Figs. 3 and 4). Mean TRTV values were in the range of − 4 to + 14% in all tissues, except for the retina (Table 1). Mean TRTV values were positive in some tissues (i.e., higher *k*_E_ in the retest scan) and negative in other tissues (i.e., lower *k*_E_ in the retest scan).

We performed dosimetry calculations on PET data from all subjects (Table 2). In agreement with the predominantly urinary excretion of activity (Fig. 3), the urinary bladder and the kidneys received the highest absorbed doses for both sexes. Effective doses were 4.67 ± 0.18 µSv/MBq for men and 4.55 ± 0.18 µSv/MBq for women.

**Table 2:** Absorbed doses for selected organs/tissues and effective doses resulting from [^11^C]BMP in male and female subjects

	Male ^a^	Female ^b^
**Organ**	Absorbed organ dose per unit activity (µGy/MBq)	
Adrenals	5.41 ± 0.48	4.59 ± 0.43
Gall bladder wall	4.05 ± 0.36	4.67 ± 0.41
Kidneys	15.09 ± 2.26	12.40 ± 1.81
Liver	1.39 ± 0.10	1.44 ± 0.11
Lymphatic nodes	4.35 ± 0.34	4.39 ± 0.33
Pancreas	5.16 ± 0.47	5.34 ± 0.50
Rectosigmoid colon wall	6.92 ± 0.85	7.63 ± 0.87
Thymus	3.13 ± 0.39	3.21 ± 0.39
Ureters	6.37 ± 0.55	6.24 ± 0.54
Urinary bladder wall	27.99 ± 3.64	28.35 ± 3.59
	Effective dose per unit activity (µSv/MBq)	
	4.67 ± 0.18	4.55 ± 0.18

^a^*n* = 7 men

^b^*n* = 6 women

## Discussion

The use of LAFOV PET/CT [23] enabled us to assess, at a whole-body, multi-tissue level, the function of MRPs, which have a widespread tissue distribution and necessitate quantitative dynamic PET measurements for their assessment. This will allow for human translation of results from previous mouse studies, in which the axial FOV of the employed small-animal PET scanner permitted a whole-body quantification of tissue MRP function [9, 10, 13].

A prerequisite to visualise the function of tissue MRPs with [^11^C]BMP is its efficient conversion into the corresponding GSH conjugate [^11^C]MPG. This conversion is mediated by GSTs which have a widespread tissue distribution. Rapid and almost quantitative conversion (i.e., within 5–15 min after radiotracer injection) of [^11^C]BMP into [^11^C]MPG has been demonstrated in the mouse brain and lungs [7–9] and in the rat lungs [12]. We analysed in our study plasma samples as a surrogate for tissue samples to estimate the extent of [^11^C]BMP conversion in human tissues (Fig. 2a). We found that 63 ± 5% of total radioactivity in plasma represented the radiolabelled GSH conjugate [^11^C]MPG at 5 min after radiotracer injection. It appears likely that GSH conjugation of [^11^C]BMP was already completed at the 5 min time point and that the remaining unconverted [^11^C]BMP in plasma represented the plasma protein-bound fraction of [^11^C]BMP, which is not able to distribute into erythrocytes, where GSH conjugation presumably occurs. The decline in the percentage of [^11^C]MPG in plasma at later time points (Fig. 1b) may be related to its faster plasma clearance relative to the other radiolabelled species. It is not known, however, whether the remaining unconverted [^11^C]BMP is able to distribute from plasma to tissues, which could affect the measurement of tissue MRP function using the *k*_E_ parameter. Therefore, in future work attempts will be made to develop a full kinetic model for [^11^C]BMP, which will benefit from the presence of large blood vessels within the FOV of the LAFOV PET/CT scanner for the measurement of an image-derived arterial input function.

For quantification of tissue MRP function we derived, in analogy to previous rodent studies [7–9], the *k*_E_ parameter from the tissue TACs. Knockout of the *Abcc1* gene or pharmacological inhibition of MRPs with MK571 led to significant decreases of *k*_E_ in the mouse brain and lungs [7–11]. In the brain, MRP1 is predominantly expressed in brain parenchymal cells (e.g., astrocytes and neurons) and in choroid plexus epithelial cells with lower expression levels in brain capillary endothelial cells (BCECs) [24]. Okamura et al. demonstrated, by intracranial injection of [^11^C]MPG into wild-type and *Abcc1*^*(−/−)*^ mice, that MRP1 function is not the rate limiting step in the elimination of [^11^C]MPG from the mouse brain [15]. Experiments in mice lacking organic anion transporter 3 (OAT3, encoded in rodents by the *Slc22a8* gene) or multidrug resistance-associated protein 4 (MRP4, encoded in rodents by the *Abcc4* gene) showed that the elimination of [^11^C]MPG across the mouse BBB is mediated by OAT3 and MRP4, which are localised in the basolateral (brain-facing) and luminal (blood-facing) membranes of BCECs, respectively [15]. An important finding of our study is that, in contrast to the rapid elimination of [^11^C]BMP-derived radioactivity from the mouse brain (*k*_E_: 1.48 ± 0.06 h^− 1^) [9], radioactivity was very slowly eliminated from the human brain with 30–45 times lower *k*_E_ values in humans (Table 1). This strongly suggests pronounced species differences in the expression of membrane transporters that can efflux GSH conjugates such as [^11^C]MPG across the BBB. While OAT3 and MRP4 could be quantified in mouse BCECs with targeted proteomics [25], they were below the limit of detection in human BCECs [26]. It is not certain whether species differences in MRP1 expression contributed to the slow elimination of [^11^C]BMP-derived radioactivity from the human brain, since available immunohistochemistry data indicate that MRP1 is expressed in the human brain [24]. Our observation is in good agreement with results for the brain perfusion single photon emission computed tomography (SPECT) tracer [^99m^Tc]Tc-ECD (i.e., ^99m^Tc complex with *N*,*N*’-1,2-ethylenediylbis-L-cysteine) [27]. [^99m^Tc]Tc-ECD is converted inside the brain into an acid metabolite, which is a substrate of OAT3 and trapped in the human brain while being quickly eliminated from the mouse brain. We also analysed the choroid plexus, in which MRP1 is expressed in the basolateral (blood-facing) membrane of epithelial cells [28], and the retina, in which MRP1 is expressed in the cell membrane of the retinal pigment epithelium forming the outer blood-retina barrier [29]. Interestingly, both tissues showed a considerably faster radioactivity elimination as compared to the brain (Table 1), supporting the presence of some efflux mechanism for [^11^C]BMP-derived radioactivity. It remains to be determined in future studies involving administration of an MRP1 inhibitor whether the kinetics of [^11^C]BMP-derived radioactivity in the human brain are dependent on MRP1 function and whether the observed regional and sex differences are caused by differences in MRP1 function or by other factors, such as differences in GST activity or GSH content.

Apart from central tissues, MRP1 is abundantly expressed in the basolateral membrane of pulmonary epithelial cells [30]. It has been shown that [^11^C]BMP PET can measure MRP1 function in the rodent lungs, both after i.v. administration [8–10, 13] and after intratracheal aerosolisation [12, 14]. As opposed to the pronounced species differences observed in the brain kinetics of [^11^C]BMP-derived radioactivity, the elimination rate of radioactivity from the human lungs was in similar range as in mice (*k*_E_, humans: 0.875 ± 0.095 h^− 1^, mice: 1.52 ± 0.10 h^− 1^) [9]. Intriguingly, we observed significant sex differences in the lungs with lower *k*_E_ values in women than in men (Table 1). This agrees well with quantitative proteomics data, which revealed a 30% lower expression of MRP1 in the lungs of women than of men [31], and supports that [^11^C]BMP-derived radioactivity is eliminated from the lungs by MRP1. We also analysed the myocardium, in which MRP1 is expressed in the sarcolemmal membrane of cardiomyocytes and was shown to protect the heart from doxorubicin-induced cardiotoxicity [32].

Different MRP subtypes (i.e., MRP2 and MRP4) are abundantly expressed in excretory organs (i.e., the kidneys and the liver), where they mediate the urinary and biliary excretion of various drugs and their metabolites [33]. In contrast, MRP1 is only moderately or not at all expressed in the liver and kidneys. As different MRP subtypes show a considerable degree of substrate overlap, it can be expected that [^11^C]MPG is also transported by other MRP subtypes including those expressed in the kidneys and liver. In fact, experiments in *Abcc4*^*(−/−)*^ mice revealed that MRP4 contributed to the urinary excretion of [^11^C]BMP-derived radioactivity in mice [9]. To assess renal and hepatic MRP function with [^11^C]BMP PET we included the kidneys, the liver, and the urinary bladder into our analysis. The gall bladder was not always clearly visible on the PET images and could therefore not be analysed in all subjects. Our data revealed that [^11^C]BMP-derived radioactivity is predominantly excreted into the urine with approximately 50% of the administered activity found in the urinary bladder at the end of the PET scan (Fig. 4a). In contrast, < 5% of the administered activity was in the gall bladder at the end of the PET scan (Fig. 4b), which suggests negligible biliary excretion of [^11^C]BMP-derived radioactivity. Radio-HPLC analysis of urine samples confirmed excretion of [^11^C]MPG into the urine but also showed the presence of some other, unidentified radiolabelled species, which were not detected in plasma, suggesting that they were formed in the kidneys (Fig. 2). We found significant sex differences in *k*_E_ values in the kidneys, pointing to a higher expression of [^11^C]MPG-excreting transporters in the kidneys of women than men (Table 1). In future studies, the MRP subtype specificity of [^11^C]MPG needs to be assessed to elucidate which MRP subtypes are involved in its urinary excretion in humans.

To assess the reproducibility of [^11^C]BMP PET for measurement of tissue MRP function, we performed test-retest scans in 3 male and 3 female subjects. Test and retest scans were not performed on the same study day as this was not feasible within our study set-up. Overall, mean TRTVs were in an acceptable range for all investigated tissues, except for the retina (Table 1), despite the relatively long interval between test and retest scans (20 ± 10 days). The comparatively low reproducibility of *k*_E_ values in the retina is probably due to the small size of this structure. The between-subject variability of the TRTV values was rather large (Table 1), which may be due to physiological variability in transporter function and different time intervals between the test and retest scans in individual subjects.

We calculated human dosimetry based on the whole-body PET data. The dosimetry calculations benefitted from the rich kinetic information obtained in multiple organs of the body, which would not have been available when performing the dosimetry study on a conventional PET/CT with multiple bed positions. The organ absorbed doses did not show any conspicuous values. For both sexes, the urinary bladder and the kidneys received the highest absorbed doses (Table 2), which was consistent with the predominantly urinary excretion of [^11^C]BMP-derived radioactivity (Fig. 4). The effective doses (Table 2) were comparable to human effective doses extrapolated from mouse PET data, which had been included in the IMPD of [^11^C]BMP [18]. The dosimetry of [^11^C]BMP was in the typical range of other ^11^C-tracers (average effective dose of 77 different ^11^C-tracers: 5.2 ± 1.7 µSv/MBq, range: 3.2–14.1 µSv/MBq) [34]. An injected activity of 400 MBq thus corresponds to an effective dose of 1.87 mSv in men and 1.82 mSv in women, which is well below the limit of 10 mSv for studies in healthy volunteers aged less than 50 years [35] and will permit multiple [^11^C]BMP PET scans in a single subject. Future studies will include a validation of [^11^C]BMP for measurement of tissue MRP function in humans by performing LAFOV PET/CT scans without and with pre-treatment with an MRP-inhibiting drug, for which the here reported test-retest data will be useful.

In summary, the advent of LAFOV PET/CT offers the unprecedented opportunity to assess molecular targets relevant for drug disposition in humans at a whole-body, multi-tissue level. This innovative research approach holds enormous potential for evaluating the disposition of radiolabelled drugs in humans in health and disease, as well as for investigating factors that may alter drug disposition (e.g., drug-drug interactions). Based on our current data, [^11^C]BMP is a safe radiotracer that will, in combination with LAFOV PET/CT, potentially allow the comprehensive assessment of MRP function across multiple tissues in the human body.

## Electronic supplementary material

Below is the link to the electronic supplementary material.

Supplementary Material 1

## Data Availability

The datasets generated during and analysed during the current study are available from the corresponding author on reasonable request (OL).
