# Supplementary material for: First-in-human evaluation of 6-bromo-7-[11C]methylpurine, a PET tracer for assessing the function of multidrug resistance-associated proteins in different tissues
Source: Eur J Nucl Med Mol Imaging. 2024 Jul 26;51(13):3900–11. doi: 10.1007/s00259-024-06851-2 (PMC11527933; doi:10.1007/s00259-024-06851-2)
Supplement: Supplementary file 1 — Supplementary Material 1 [file 259_2024_6851_MOESM1_ESM.docx]

**Supplementary Information**

**First-in-human evaluation of 6-bromo-7-[^11^C]methylpurine, a PET tracer for assessing the function of multidrug resistance-associated proteins in different tissues**

Severin Mairinger^1,2^, Matthias Jackwerth^1^, Zacharias Chalampalakis^3^, Ivo Rausch^3^, Maria Weber^1^, Michael Wölfl-Duchek^1,2^, Lena Pracher^1^, Lukas Nics^2^, Jens Pahnke^4,5,6,7,8^, Werner Langsteger^2^, Marcus Hacker^2^, Markus Zeitlinger^1^, Oliver Langer^1,2^

^1^ Department of Clinical Pharmacology, Medical University of Vienna, Vienna, Austria

^2^ Department of Biomedical Imaging and Image-guided Therapy, Medical University of Vienna, Vienna Austria

^3^ QIMP Team, Center for Medical Physics and Biomedical Engineering, Medical University of Vienna, Vienna, Austria

^4^ Translational Neurodegeneration Research and Neuropathology Lab, Department of Clinical Medicine (KlinMed), Medical Faculty, University of Oslo, Oslo, Norway

^5^ Section of Neuropathology Research, Department of Pathology, Clinics for Laboratory Medicine (KLM), Oslo University Hospital, Oslo, Norway

^6^ Institute of Nutritional Medicine (INUM) and Lübeck Institute of Dermatology (LIED), University of Lübeck and University Medical Center Schleswig-Holstein, Lübeck, Germany

^7^ Department of Pharmacology, Faculty of Medicine and Life Sciences, University of Latvia, Rīga, Latvia

^8^ School of Neurobiology, Biochemistry and Biophysics, The Georg S. Wise Faculty of Life Sciences, Tel Aviv University, Tel Aviv, Israel

**Supplementary Table 1** Percentages of the three radiolabelled species in plasma of male and female subjects at different time points after radiotracer injection ([^11^C]MPG = radiolabelled glutathione conjugate, [^11^C]BMP = parent tracer, [^11^C]U = unidentified lipophilic radiolabelled species).

|  | **5 min** | **20 min** | **40 min** |
| --- | --- | --- | --- |
|  | % of total radioactivity (mean ± SD) | | |
| **Male** ^a^ |  |  |  |
| [^11^C]MPG | 60 ± 3 | 43 ± 3 | 37 ± 3 |
| [^11^C]BMP | 30 ± 4 | 30 ± 5 | 31 ± 6 |
| [^11^C]U | 4 ± 1 | 19 ± 4 | 22 ± 5 |
| **Female** ^b^ |  |  |  |
| [^11^C]MPG | 66 ± 8 | 46 ± 8 | 38 ± 6 |
| [^11^C]BMP | 25 ± 8 | 26 ± 8 | 25 ± 10 |
| [^11^C]U | 3 ± 2 | 23 ± 3 | 30 ± 3 ^*^ |

^a^ *n* = 7 men

^b^ *n* = 5 women, one subject was excluded due to insufficient plasma quantities

^*^ Significantly different from men (*p* ≤ 0.05; two-sided, unpaired t-test)


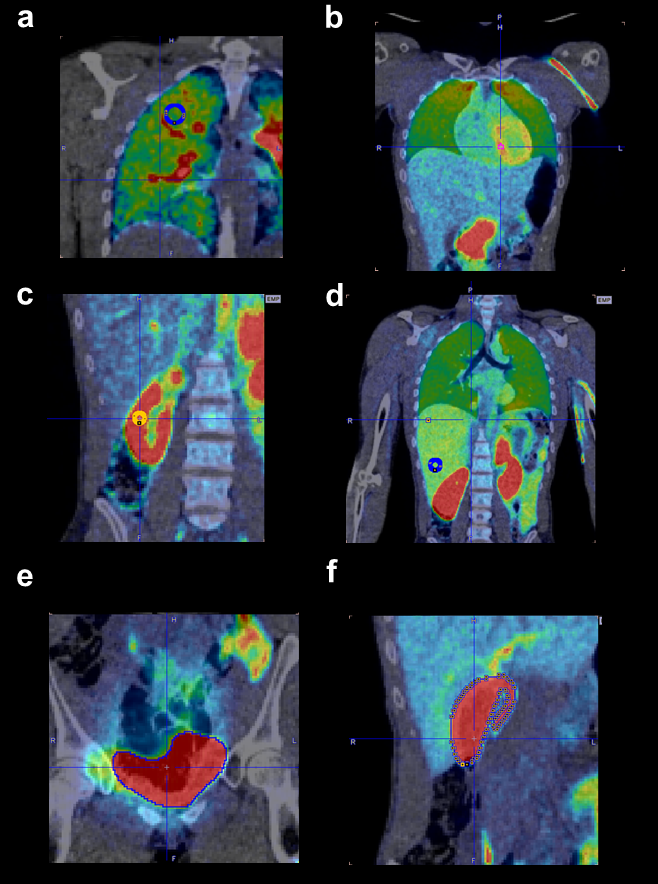


**Supplementary Figure 1.** Examples for the manually outlined VOIs in peripheral tissues on co-registered PET/CT images in coronal planes (**a**: right lung; **b**: myocardium; **c**: right kidney cortex; **d**: liver; **e**: urinary bladder; **f**: gall bladder). For the lung (**a**) and the liver (**d**), only one of three outlined spherical VOIs is visible in the displayed planes.


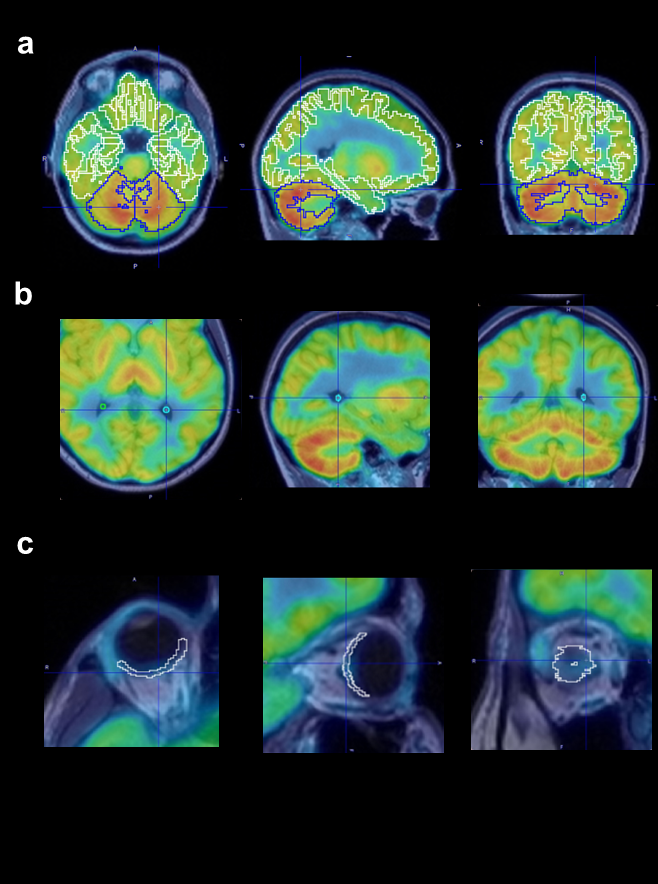


**Supplementary Figure 2.** Examples for the outlined VOIs in central tissues on co-registered PET/MR images in axial, sagittal and coronal planes (**a**: blue = cerebellar grey matter; white = cortical grey matter; **b**: choroid plexus; **c**: retina). VOIs shown in panel **a** were automatically outlined using the N30R83 brain atlas, while VOIs shown in panels **b** and **c** were manually outlined.





**Supplementary Figure 3.** *k*_E_ values in central MRP-expressing tissues, i.e., cortex (**a**), cerebellum (**b**), choroid plexus (**c**), and retina (**d**), for test and retest scans in male (*n* = 3, blue triangles) and female subjects (*n* = 3, red circles). ns, not significant; two-sided, paired t-test.





**Supplementary Figure 4.** *k*_E_ values in peripheral MRP-expressing tissues, i.e., right lung (**a**), myocardium (**b**), right kidney cortex (**c**), and liver (**d**), for test and retest scans in male (*n* = 3, blue triangles) and female subjects (*n* = 3, red circles). ns, not significant; two-sided, paired t-test.
